# Supplementary material for: Multi‐dataset identification of innovative feature genes and molecular mechanisms in keratoconus
Source: J Cell Mol Med. 2024 Sep 19;28(18):e70079. doi: 10.1111/jcmm.70079 (PMC11412914; doi:10.1111/jcmm.70079)
Supplement: Supplementary file 2 — Material S2. [file JCMM-28-e70079-s002.zip › 7.GSEA analysis/1.ARL11/KEGG_GLYCOSAMINOGLYCAN_DEGRADATION.html]

Details for gene set KEGG\_GLYCOSAMINOGLYCAN\_DEGRADATION[GSEA]

|  || Dataset | ARL11.group.cls#h\_versus\_l |
| Phenotype | group.cls#h\_versus\_l |
| Upregulated in class | h |
| GeneSet | KEGG\_GLYCOSAMINOGLYCAN\_DEGRADATION |
| Enrichment Score (ES) | 0.27313238 |
| Normalized Enrichment Score (NES) | 0.85076916 |
| Nominal p-value | 0.6323232 |
| FDR q-value | 1.0 |
| FWER p-Value | 1.0 |
Table: GSEA Results Summary

  

Fig 1: Enrichment plot: KEGG\_GLYCOSAMINOGLYCAN\_DEGRADATION      
 Profile of the Running ES Score & Positions of GeneSet Members on the Rank Ordered List

  

| SYMBOL | TITLE | RANK IN GENE LIST | RANK METRIC SCORE | RUNNING ES | CORE ENRICHMENT || 1 | HS3ST3A1 | na | 211 | 0.572 | 0.2731 | Yes |
| 2 | HPSE2 | na | 7854 | 0.095 | 0.0995 | No |
| 3 | HGSNAT | na | 8945 | 0.076 | 0.1050 | No |
| 4 | SGSH | na | 9617 | 0.066 | 0.1177 | No |
| 5 | IDUA | na | 11290 | 0.044 | 0.0910 | No |
| 6 | NAGLU | na | 11534 | 0.041 | 0.1040 | No |
| 7 | HYAL3 | na | 11538 | 0.041 | 0.1240 | No |
| 8 | HYAL2 | na | 13595 | 0.022 | 0.0756 | No |
| 9 | GNS | na | 13966 | 0.019 | 0.0742 | No |
| 10 | GALNS | na | 14748 | 0.013 | 0.0581 | No |
| 11 | HEXA | na | 16191 | 0.002 | 0.0177 | No |
| 12 | GUSB | na | 16664 | -0.001 | 0.0046 | No |
| 13 | GLB1 | na | 18269 | -0.012 | -0.0357 | No |
| 14 | HYAL1 | na | 19473 | -0.020 | -0.0605 | No |
| 15 | HYAL4 | na | 21719 | -0.037 | -0.1071 | No |
| 16 | HEXB | na | 22982 | -0.050 | -0.1192 | No |
| 17 | IDS | na | 25168 | -0.078 | -0.1439 | No |
| 18 | HPSE | na | 28392 | -0.141 | -0.1681 | No |
| 19 | SPAM1 | na | 30817 | -0.215 | -0.1333 | No |
| 20 | HS3ST3B1 | na | 31654 | -0.246 | -0.0375 | No |
| 21 | ARSB | na | 31958 | -0.258 | 0.0798 | No |
Table: GSEA details [plain text format]

  

Fig 2: KEGG\_GLYCOSAMINOGLYCAN\_DEGRADATION      
 Blue-Pink O' Gram in the Space of the Analyzed GeneSet

  

Fig 3: KEGG\_GLYCOSAMINOGLYCAN\_DEGRADATION: Random ES distribution      
 Gene set null distribution of ES for **KEGG\_GLYCOSAMINOGLYCAN\_DEGRADATION**
